# UV-Cured Poly(Siloxane-Urethane)-Based Polymer Composite Materials for Lithium Ion Batteries—The Effect of Modification with Ionic Liquids

**DOI:** 10.3390/ma13214978

**Published:** 2020-11-05

**Authors:** Janusz Kozakiewicz, Jarosław Przybylski, Bartosz Hamankiewicz, Krystyna Sylwestrzak, Joanna Trzaskowska, Michal Krajewski, Maciej Ratyński, Witold Sarna, Andrzej Czerwiński

**Affiliations:** 1 Department of Polymer Technology and Processing, Łukasiewicz Research Network—Industrial Chemistry Institute, 01-793 Warsaw, Poland; jaroslaw.przybylski@ichp.pl (J.P.); krystyna.sylwestrzak@ichp.pl (K.S.); joanna.trzaskowska@ichp.pl (J.T.); witold.sarna@ichp.pl (W.S.); 2 Faculty of Chemistry, University of Warsaw, 02-093 Warsaw, Poland; mkrajewski@chem.uw.edu.pl (M.K.); mratynski@chem.uw.edu.pl (M.R.); aczerw@chem.uw.edu.pl (A.C.)

**Keywords:** poly(siloxane-urethane)s, lithium ion batteries, separators, composite polymer materials, ionic liquids, gel polymer electrolytes, UV-curing

## Abstract

The results of studies on the synthesis and characterization of conductive polymer composite materials designed as potential separators for lithium ion batteries are presented. The conductive polymer composites were prepared from UV-cured poly(siloxane-urethanes)s (PSURs) containing poly(ethylene oxide) (PEO) segments and modified with lithium salts and ionic liquids (ILs). The most encouraging results in terms of specific conductivity and mechanical properties of the composite were obtained when part of UV-curable PSUR prepolymer was replaced with a reactive UV-curable IL. Morphology of the composites modified with ILs or containing a standard ethylene carbonate/dimethyl carbonate mixture (EC/DMC) as solvent was compared. It was found that the composites showed a two-phase structure that did not change when non-reactive ILs were applied instead of EC/DMC but was much affected when reactive UV-curable ILs were used. The selected IL-modified UV-cured PSUR composite that did not contain flammable EC/DMC solvent was preliminarily tested as gel polymer electrolyte and separator for lithium ion batteries.

## 1. Introduction

Studies on the synthesis and characterization of poly(siloxane-urethane)s (PSURs) containing poly(ethylene oxide) (PEO) segments as potential polymer composite materials for application in lithium ion batteries were conducted in the past in the authors’ laboratory, and both moisture-cured and UV-cured PSUR systems were investigated [1,2]. In this paper, the results of studies on using ionic liquids (ILs) as modifiers for such UV-cured PSUR composite materials that at the same time could play the role of separators and gel polymer electrolytes in lithium ion batteries are described.

Various UV-cured crosslinked gel polymer electrolytes have been studied so far, primarily including polymerized di- or triacrylate or di- or trimethacrylate esters of PEO with doped lithium salts [3,4,5,6,7,8,9,10,11]. Similar systems based on UV-crosslinked siloxane diacrylate monomers [12,13] or polyurethane-acrylate [14,15] have also been investigated. However, in most of these works, flammable solvents were used, including mainly ethylene carbonate (EC), propylene carbonate (PC), or a standard flammable 1/2 *v*/*v* mixture of EC with dimethyl carbonate (DMC) referred to as EC/DMC in this paper. Therefore, following earlier studies [16,17], the possibility of replacing these flammable solvents in crosslinked (usually UV-cured) polymer systems designed for lithium ion batteries with non-flammable ILs was investigated more deeply [18,19,20,21,22,23,24,25,26,27]. Specifically, application of the concept of single-ion conducting polymer electrolytes by synthesizing a polyurethane based on PEO and specifically designed IL monomer seemed to be quite promising [24].

According to the generally accepted definition [28], ILs are organic-organic or organic-inorganic salts that are liquids at temperatures below 100 °C. ILs have been widely used, mainly as catalysts and solvents in the synthesis of organic compounds [29] and polymers [30], including in mixtures with supercritical CO_2_ [31], vulcanization accelerators for rubber [32], and also as electrolytes [33,34,35,36,37,38], specifically for lithium batteries [39,40,41,42,43,44,45,46,47]. A comprehensive literature review concerning ILs in polymer systems was published [48], and the role of ILs in lithium ion batteries was explained in detail in another review [49]. It was stated there that owing to the enormous possibilities of ionic liquids they can be used to design novel types of electrolytes for a new generation of lithium ion batteries.

ILs can be considered as components of polymer composite systems applied in lithium ion batteries, since they are conductive, non-flammable, and non-volatile, so using them can eliminate or at least significantly diminish the content of volatile and flammable solvents in such systems. Moreover, the presence of ILs in the composite may plasticize it and thus help to solve the problem of the increase in glass transition temperature of the polymeric composite material with an increase in lithium salt content, which results in the reduction of PEO segment mobility. As a result, it is not possible to achieve high conductivity of the composite above certain concentration of lithium salt [50]. A conductivity mechanism in lithium ion batteries containing both lithium salts and ILs as electrolytes was described in [51]. More recently, a comparison of electrochemical properties of lithium ion batteries containing ILs and conventional solvents was made [52]. The cell containing IL exhibited good capacity retention and rate capability during 100 cycles, while rapid capacity fading was found for the corresponding cell with the organic electrolyte.

In practice, only ILs that are liquids at or below room temperature (RT) and thus are known as “room temperature ionic liquids” (RTILs) can be applied in lithium ion batteries [39,53]. RTILs that are most often offered for this purpose contain alkyl-substituted quaternary ammonium, pyrrolidinium or imidazolium cations, and trifluoromethanesulfonium (CF_3_SO_3_^−^) or bis(trifluoromethanesulfonyl)imide ((CF_3_SO_2_)_2_N^−^) anions, which are commonly called TFS or TFSI anions. In lithium ion batteries, solutions of lithium salts in RTILs are usually applied, so in fact new RTILs of a general formula [Li^+^]_m_[A^+^]_n_[X^−^]_m+n_ are formed. In a review published in 2010 [39], a number of such RTIL-lithium salt combinations were listed as suitable electrolytes for lithium ion batteries with different types of electrodes, C/LiCoO_2_ and C/Li being the most frequently mentioned. The most widely investigated systems based on RTILs and lithium salts contained high molecular weight (MW) PEOs [54,55,56,57,58]. RTILs were also used in mixtures with PEO and alkali metal salts [59,60], and the synthesis of pyrrolidinium-based poly(ionic liquid) electrolytes with PEO side chains was also described [61]. There were attempts to synthesize RTILs containing imidazolium cation N-substituted with a PEO chain terminated with a methoxy group [56], or even lithium and PEO containing RTILs with anion of a structure Li{[CH_3_(OCH_2_CH_2_)_n_O]_3_BC_4_H_9_} [62]. Highly conductive RTILs, which did not contain lithium, were obtained through the photopolymerization of monomers in the presence of high boiling solvent (tetramethylsulfone, bp = 287 °C), which remained in the film after polymerization process and acted as plasticizer [63]. The idea of applying the photopolymerization process in the preparation of polymer gel electrolytes for lithium ion batteries was used in practice also by other investigators [16,17,21,22,23,24,25,26,27]. If the RTIL could not polymerize, the process of UV curing that led to film formation was accomplished due to the creation of free radicals on PEO chain [26]. However, ILs containing unsaturated bonds and thus capable for polymerization were also investigated as monomers or co-monomers in polymer synthesis [64]. Several such polymerizable ILs were described in a general review comprising various applications of ILs [30].

Recently, the possibility of applying ILs containing both ionic liquid-like moieties and polymer frameworks in lithium ion batteries was investigated, and it was found that such systems behaved very well and thus might represent a novel promising direction in this field [65]. Another review that has been very recently published concerned ILs-containing polymer/inorganic hybrid electrolytes designed for lithium ion batteries [66].

In general, most of the safety issues faced in lithium batteries result from liquid electrolyte presence in lithium battery systems. Lithium plating during overcharge and uncontrolled SEI (solid electrolyte interphase) growth on the negative electrode can result in internal short-circuiting of the cell [67]. Moreover, high flammability of liquid electrolytes can be responsible for cell explosion during thermal runaway in case of battery unsealing [65]. Those disadvantages can be mitigated by introducing solid-state electrolytes into the battery system, increasing their safety characteristic, and prolonging the cell operational lifetime [66]. For example, a polymeric ionic liquid electrolyte implemented into a Li/LiFePO_4_ lithium cell, which delivered ca. 150 mAh g^−1^ specific capacity at RT and 0.1 C rate was described [68]. After 100 consecutive charge/discharge processes, the cell retained 97.7% of its initial capacity.

The high temperature performance of lithium battery systems containing solid-state electrolytes was also considered. Polymeric ionic liquid electrolyte containing LiTFSI as lithium salt and lithium-titanium-aluminum phosphate as inorganic filler were tested in Li/LiFePO_4_ cell at 60 °C [69]. At a current density of 1 C, the cell delivered 141.3 mAh g^−1^ specific capacity and showed the capacity retention of 96.4% after 250 consecutive charge/discharge cycles. The use of polymeric ionic liquid electrolytes in lithium battery systems was also evaluated in other battery chemistries, including Li_4_Ti_5_O_12_/LiMn_2_O_4_ and HC (hard carbon)/LiMn_2_O_4_ [70] Li_4_Ti_5_O_12_/LiCoO_2_ [71] or C (graphite)/LiNi_0.5_Mn_0.3_Co_0.2_ O_2_ [72] showing their capabilities as ion transporting media in examined systems.

In this paper we present the results of our studies on the synthesis and characterization of polymer composite materials consisting of UV-curable PSUR, lithium salts, and ILs, both non-reactive and reactive, that were patented [73,74] and were preliminarily tested as gel polymer electrolytes and separators in lithium batteries.

## 2. Materials and Methods

### 2.1. Materials

**Figure 1:**
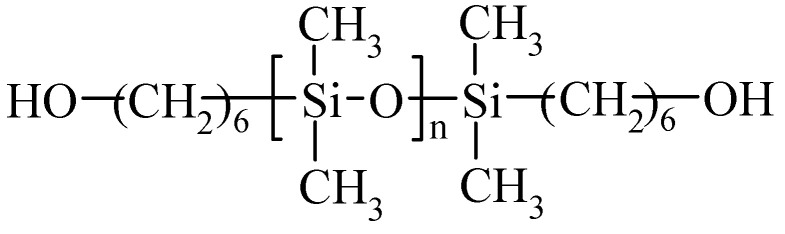
Structure of polysiloxane diol (SIL) used in the study. n = 30.

Isophorone diisocyanate (IPDI) was obtained from Evonik Degussa Polska sp. z o. o. (Warsaw, Poland), PEO with 1 kDa molecular weight was obtained from Merck Polska sp. z o. o. (Warsaw, Poland) and polysiloxane diol (SIL) with a structure shown in Figure 1 (Tegomer 2311) was obtained from Tego Chemie (presently Evonik, Essen, Germany). Dimethyl carbonate (DMC) and ethylene carbonate (EC) solvents, unsaturated diol (Cis-2-butene-1,4-diol), metallic lithium, as well as lithium salts (LiTFS-LiCF_3_SO_3_ and LiTFSI-LiN(CF_3_SO_2_)_2_) were obtained from Sigma-Aldrich Polska sp. z o. o. (Poznań, Poland). Dibutyltin dilaurate catalyst (DBTL) was obtained from POCH (Gliwice, Poland). UV curing initiator Irgacure 2022 was obtained from Sigma-Aldrich Polska sp. z o.o. (Poznań, Poland).

Selection of ILs applied in the study was made based on the following criteria:

- The same anion as the anion in lithium salt, i.e., −CF_3_SO_3_^−^ (TFS) and (CF_3_SO_2_)_2_N^−^ (TFSI)
- Low viscosity, optionally also at low temperatures, and the melting point below −30 °C [75]
- Good compatibility with other components of the composite, both before and after curing, including capability to dissolve lithium salt
- Good specific conductivity at room temperature (around 10^−3^ Scm^−1^)
- No effect on surface tension of the composition before curing (to avoid defects of the film when the composition is cast on the substrate for curing)
- Minimum (optionally zero) water content
- Low price (if commercially available) or low production cost (if it is to be synthesized)

Apart from conventional ILs, we tested in our system the ILs that potentially could be built in the final structure of the polymer constituting the composite. In the preliminary experiments, both ILs containing double bonds [30] or ILs containing hydroxyl groups, such as, for example, IL containing hydroksyethyl-3-methylimidazolium cation and BF_4_^−^ anion [76], were tested. However, the latter option was eliminated as it did not allow for films with reasonable mechanical properties to be obtained.

Names, substantial properties, and structures of ILs finally used in the study are shown in Table 1. ILs 1–4 were synthesized in the Łukasiewicz Research Network—Industrial Chemistry Institute, Warsaw, Poland, and ILs 5 and 6 were obtained from Sigma-Aldrich.

In order to acquire more information on the potential behavior of the ILs in the PSUR-based composites prepared in the course of the study, the thermal properties of these ILs were examined by Differential Scanning Calorimetry (DSC). The results are presented in Table 2.

### 2.2. Methods

#### 2.2.1. Synthesis of PSUR Prepolymer

SIL and PEO were dehydrated by heating for 2 h at 120 °C under vacuum. The polyaddition reaction of SIL and PEO (3/1) with IPDI (NCO/OH = 2.5/1) was carried out at 80 °C in a glass reactor 0.75 dm^3^ equipped with anchor type agitator and inert gas inlet in the presence of DBTL catalyst until the band corresponding to OH groups (3500–3200 cm^−1^) was not detected by Fourier Transformed Infrared Spectroscopy (FTIR) Free NCO content of the resulting prepolymer was determined by titration.

#### 2.2.2. Preparation of PSUR-Based UV-Cured Composites

**Figure 2:**
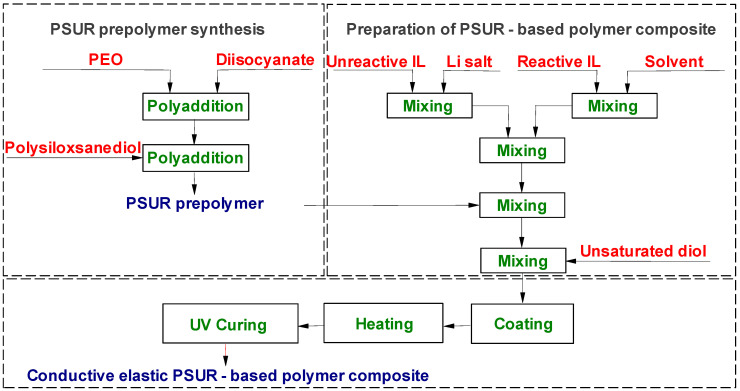
Process of preparation of ILs-containing poly(siloxane-urethane) (PSUR)-based conductive polymer composite.

PSUR-based UV-cured composites containing ILs were obtained in a two-step process as shown in Figure 2. Step I: PSUR prepolymer synthesis. Step II: Preparation of PSUR-based polymer composite.

**Figure 3:**
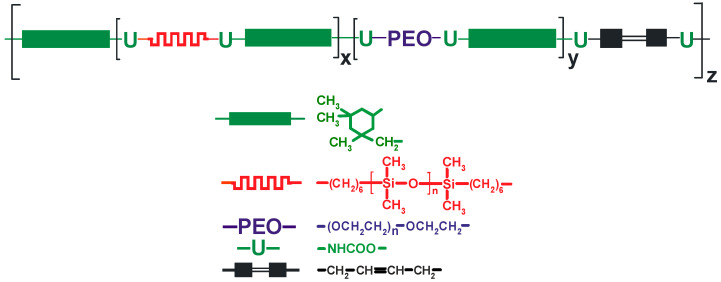
Structure of UV-curable PSUR.

The NCO-terminated poly(siloxane-urethane) prepolymer (NCO content 4.66%) obtained as described in Section 2.2.1 was mixed with a suitable quantity of unsaturated diol, UV initiator, and 1 M lithium salt solution in IL in a mixture of different ILs or (in some experiments) in a mixture with IL and EC/DMC solvent. The composition thus obtained that contained UV-curable PSUR (structure shown in Figure 3) was cast onto polytetrafluoroethylene (PTFE)-coated steel plates using the applicator with appropriate gap size and heated in an MBraun dry chamber (M. Braun Inertgas-Systeme GmbH, Garching, Germnany) at fixed temperature (120 °C) over a fixed time period (11.5 min). These heating parameters were optimized in the preliminary investigations. In some experiments, lithium salt solution in an EC/DMC = 1/2 (*v*/*v*) mixture or in DMC only was used in order to compare the properties of composites produced with and without ILs. The film obtained after heat-curing only was very weak and sticky, so it was not fully cured. Next, the plates with that film were placed under a 450 Watt UV lamp (Trans-West GmbH sp. z o. o., Środa Wielkopolska, Poland) (at a distance of 10 cm from the lamp) and subjected to UV radiation for 120 s. After that time, 200 µ thick films (for conductivity determinations and for preliminary testing as separators for lithium batteries) or 600 µ thick films (for testing the mechanical properties and for solubility experiments) were removed from the plates.

#### 2.2.3. Testing the Properties of PSUR-Based UV-Cured Composites

##### Conductivity Determinations

Specimens (in the form of round-shaped film pieces) prepared as described in Section 2.2.2 were put between two stainless steel elements of the measurement Swagelok^®^ type steel cells and, after wetting with a drop of 30 μL of the same IL that was used in composite preparation or (in one case) solution of lithium salt in that IL and (for comparison) with a drop of 30 μL of DMC or EC/DMC = 1/2 solvent mixture, were tested using an impedance analyzer (Solartron Analytical, model SI 1260, Solartron Analytical, Leicester, UK). The impedances (R) expressed in Ohms were recalculated to specific conductivities (σ) expressed in Scm^−1^ for a given specimen thickness (l) and surface area (A) of the specimens from the Equation (1):

σ = l/R × A(1)

The mean value from 3 impedance values obtained for a minimum of 4 specimens was taken as the result.

##### Preliminary Testing the Composites as Separators in Lithium Ion Batteries

**Figure 4:**
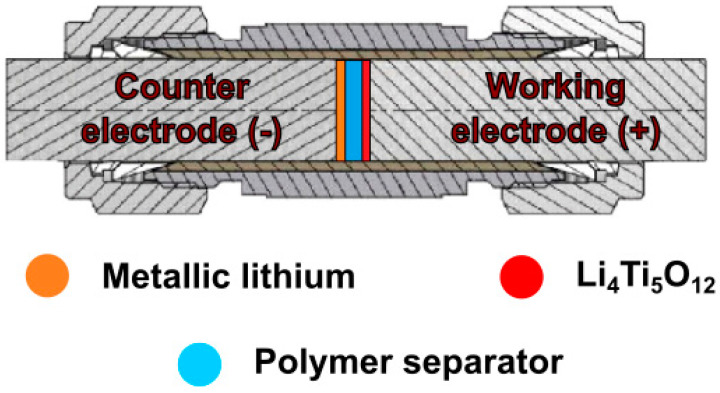
Schematic representation of lithium-ion cell in Swagelok^®^ system.

The selected composites prepared as described in Section 2.2.2 were also tested as separators in lithium-ion batteries. The films were tested in Swagelok^®^-type two-electrode systems (Figure 4), with metallic lithium as counter electrode, Li_4_Ti_5_O_12_:Vulcan^®^ XC72R (Cabot, Boston, MA, USA):PVDF (Alfa Aesar, Haverell, MA, USA) in an 8:1:1 ratio (wt) as working electrode and PSUR-based film as separator. Prior to cell assembly, the films were wetted with 10 µL of DMC. As a reference point, lithium–titanium oxide electrodes were tested in three-electrode Swagelok^®^ cells, with metallic lithium as counter and reference electrodes, Li_4_Ti_5_O_12_:Vulcan^®^ XC72R (Cabot):PVDF (Alfa Aesar) in an 8:1:1 ratio (wt) as working electrode and Celgard^®^ 2400 soaked in 1 M solution of LiTFSI in EC/DMC 1/2 (*v*/*v*). The electrochemical cells were assembled in an argon-filled glove-box (MBraun), in which H_2_O and O_2_ contaminations were kept below 0.5 ppm. Galvanostatic charge/discharge measurements were conducted on a multichannel battery tester ATLAS 1741 (Atlas-Sollich, Rębiechowo, Poland) in the voltage range of 1–3 V (two-electrode systems) at room temperature (RT), 5 °C, and 0 °C or the potential range of 1–3 V (vs. Li^+^/Li^0^) at RT for the reference sample. The charge/discharge processes were performed for 40 consecutive cycles, at 0.1 C current rate (where C equals 175 mA g^−1^) for two-electrode systems and at a 1 C rate for the reference cell. Moreover, an additional 50 cycles were executed for the polymer separator at 5 °C and current rate of 1 C.

##### Testing Thermal Properties of the Composites and ILs

DSC studies were conducted using a DSC Q2000 (TA Instruments Inc., Newcastle, DE, USA) apparatus at 20°/min heating rate according to EN ISO 11357).

##### Testing the Morphology of the Composites

Scanning Electron Microscopy with Energy Dispersive Spectroscopy (SEM/EDS) investigations were conducted using an SEM microscope (Jeol JSM-6490LV, Jeol Ltd., Tokyo, Japan).

##### Preparation of Samples for Mechanical Properties Testing and Solubility Experiments

Dumbbell-shaped specimens (62 × 10 mm) for testing the mechanical properties (Instron testing machine, Instron, High Wycombe, UK, 50 mm/min pulling rate) and round-shaped specimens (10 mm diameter) for solubility experiments were cut from the films obtained as described in Section 2.2.2. In solubility experiments, the specimens were immersed in toluene for 24 h, dried at 30 °C until there was no weight change, and percentage of fraction soluble in toluene (TSF) was determined from Equation (2).

TSF = (m_1_ − m_0_)/m_0_ × 100% (2)

where m_1_ and m_0_ are masses of the film after and before immersion, respectively.

##### FTIR Analysis

FTIR spectra were taken using KRS-5 plates consisting of thallium bromide and thallium iodide (Shimadzu) on a SPECTRUM 2000 apparatus (PerkinElmer, Inc., Waltham (MA), USA) at a resolution of 4 cm^−1^. Two scans were conducted for each spectrum.

## 3. Results and Discussion

### 3.1. Effect of Modification with Non-Reactive ILs on Specific Conductivity of the PSUR-Based UV-Cured Composites

Compositions of the PSUR-based UV-cured composites containing non-reactive ILs that were obtained in the study and the results of specific conductivity determinations are presented in Table 3.

Based on the results shown in Table 3, the following can be concluded:

(1) It appeared possible to prepare the composites without using EC or DMC solvents (compare samples A and B or D and D-D), though wetting the film for conductivity measurements with EC/DMC or DMC solvent was much more effective than wetting it with IL. That clearly suggests the crucial role of Li ion mobility for achieving higher conductivity—the mobility was much better in the composite modified with even a tiny amount of DMC or EC/DMC.

(2) Applying ILs with asymmetric structure of the pyrrolidinium or imidazolium cation (IL 5 or IL 6) allowed for better conductivity to be achieved than when ILs with highly symmetric cation structure (IL 1 or IL 2) were used. It is noteworthy that IL 5 was applied in a lithium ion experimental battery described by the other authors [77], and it was found that it had a positive effect on its electrochemical performance.

### 3.2. Effect of Modification with Non-Reactive ILs on Tg of the PSUR-Based UV-Cured Composites.

**Figure 5:**
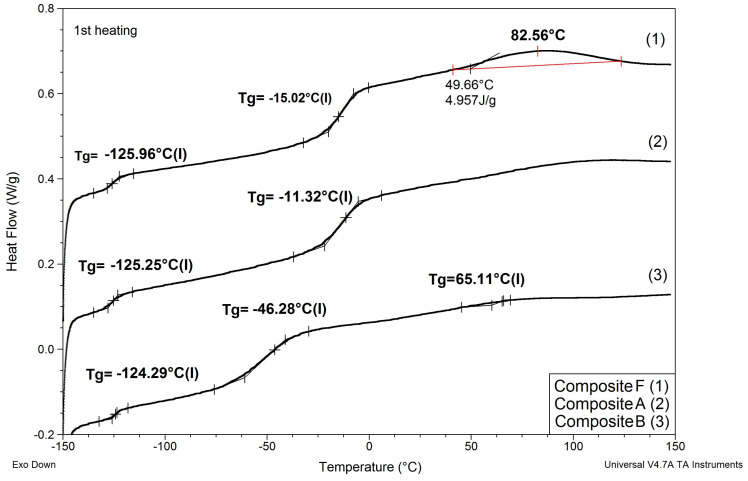
Comparison of DSC patterns obtained for selected composites prepared using EC/DMC (A), IL 5 (B), and IL/DMC (F) to dissolve lithium salt.

Results of determinations of Tg values of the composites modified with non-reactive ILs are shown in Table 4, and comparison of DSC patterns obtained for selected composites is presented in Figure 5.

Based on the results shown in Table 4 and Figure 5, the following can be concluded:

(1) Replacing EC/DMC solvent with IL did not affect Tg of silicone part (TgI) of the polymer constituting the body of the composite film. All composites showed TgI values in the narrow range −125 to −129 °C.

(2) None of the composites showed melting points of EC or ILs that would remain in the composite after curing process. This phenomenon suggests that these substances formed uniform phases with the PEO part of the polymer constituting the body of the composite film.

(3) Replacing either EC/DMC or only EC solvent with IL had a significant effect on the Tg of the PEO part (TgII) of the polymer constituting the body of the composite. Unlike TgII of the composites prepared with these solvents, TgII of the composites prepared with ILs decreased as compared to TgII of the composite prepared without lithium salt, solvents, and ILs. This phenomenon can be explained by formation of a common phase consisting of PEO, lithium salt, and IL that enabled better mobility of PEO chains.

### 3.3. Effect of Modification with Reactive ILs on Specific Conductivity and Supramolecular Structure of the PSUR-Based UV-Cured Composites

Compositions of the PSUR-based UV-cured composites containing reactive ILs that were obtained in the study and the results of specific conductivity determinations are presented in Table 5.

Based on the results shown in Table 5, the following can be concluded:

(1) Replacement of 20% of the prepolymer with reactive ILs (IL 3 or IL 4), but leaving EC/DMC as solvent resulted in slight increase (IL 3) or slight decrease (IL 4) in specific conductivity of the composite—compare sample A with G and sample A with H. However, if 80% of the prepolymer was replaced with reactive IL 3 the specific conductivity was not enhanced—compare sample K with G-C or sample K-D with G-D. The reason could be the diminishing of the concentration of PEO segments in the body of the composite film, and it is well known [78] that PEO segments are crucial for ensuring Li ion mobility.

(2) Using non-reactive IL (IL 5) instead of EC/DMC in the composite where reactive IL (IL 3) replaced 20% of prepolymer (M) led to only slight decrease in its specific conductivity. Still, relatively good specific conductivity at the level of 10^−4^ Scm^−1^ could be achieved even when the composite film was wetted with IL 5. This result was very encouraging, and therefore the composite M was selected for preliminary testing as potential separator for Li batteries.

Based on the results of conductivity determinations shown in Table 5, IL 3 was selected for further investigations.

### 3.4. Effect of Modification with Reactive ILs on Structure, Mechanical Properties and Tg of the PSUR-Based UV-Cured Composites

**Figure 6:**
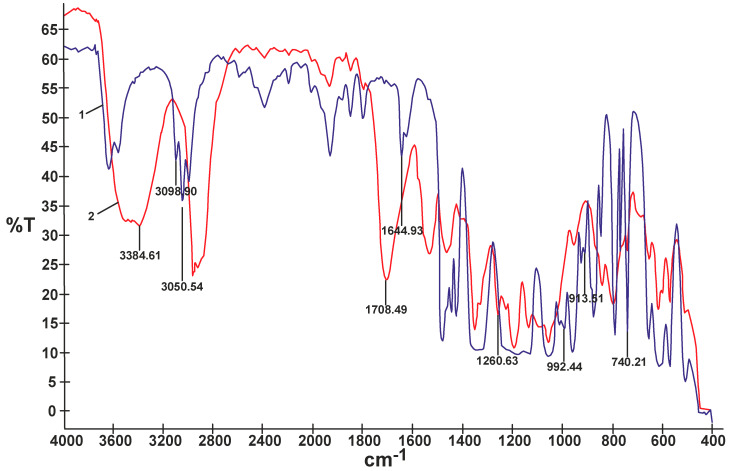
Comparison of FTIR spectra of reactive IL 3 (1) and UV-cured composite M prepared with that reactive IL 3 (2).

In contrast to the composites obtained using non-reactive ILs that produced sticky and mechanically very weak films, most of the UV-cured composites modified with reactive ILs produced less sticky transparent films showing better mechanical properties. The composite film M selected for further electrochemical studies had tensile strength equal to 2.2 MPa and elongation at break equal to 245%. The comparison of FTIR spectra of the reactive IL 3 and of the UV-cured composite M (Figure 6) proved that double bonds originating from that IL, i.e., 3090–3040 cm^−1^ and 1650–1600 cm^−1^ (C=C stretching) and 995 cm^−1^ and 915 cm^−1^ (=C–H and =CH_2_ bending), disappeared. It could be then anticipated that in the process of UV-curing, a branched or partly crosslinked structure of polymer composite material was obtained, also taking into account the results of solubility experiments—see detailed description inserted further below. Certain other bands presented in the FTIR spectrum of composite M in Figure 6, i.e., 3400–3300 cm^−1^ (N–H stretching) or 1705–1680 (C=O and N–H urethane) proved the presence of the PSUR chain. Other specific bands that could be identified in that spectrum are Si–CH_3_ (bending) at 1260 cm^−1^ from the polysiloxane chain structure and 907 cm^−1^ (C–F_3_ from TFSI anion).

Solubility experiments confirmed that a partly crosslinked structure of the composite M prepared using the reactive IL 3 was actually obtained after UV-curing. While the percentage of toluene-soluble fraction (TSF) in composites prepared with non-reactive ILs was in the range of 20%, that percentage was much lower (ca. 8%) when reactive ILs were applied. It is also worth noting that the TSF value in all composites after thermal curing, but still before UV-curing, was at the level of ca. 30%.

**Figure 7:**
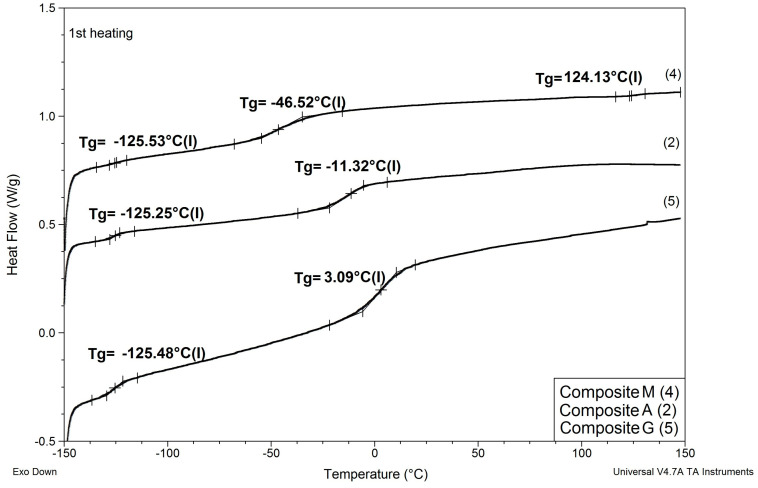
Comparison of DSC patterns obtained for composite A prepared without any IL and composites G and M, where 20% of PSUR prepolymer was replaced with reactive IL 3, and lithium salt was dissolved, respectively, in EC/DMC (like in A) and in non-reactive IL 5.

**Figure 8:**
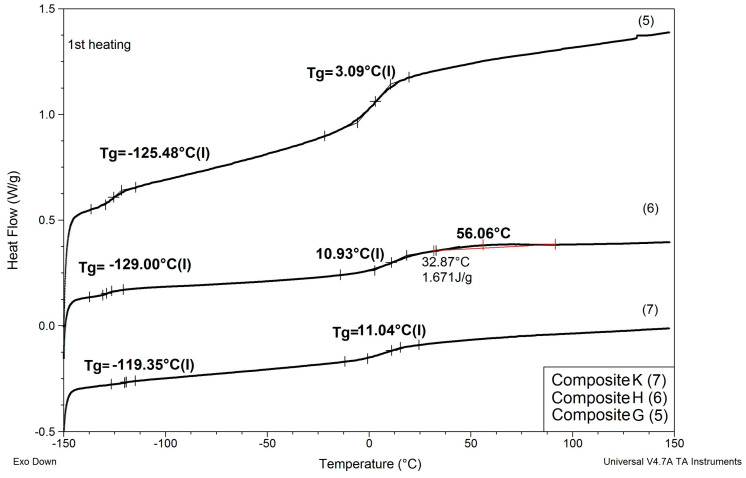
Comparison of DSC patterns obtained for composites G and H (prepared with reactive IL 3 and IL 4, respectively, replacing 20% of PSUR prepolymer), and composite K (prepared with reactive IL 3 replacing 80% of prepolymer).

Results of determinations of Tg values of the composites modified with reactive ILs are shown in Table 6, and comparisons of DSC patterns obtained for selected composites are presented in Figure 7 and Figure 8.

**Figure 9:**
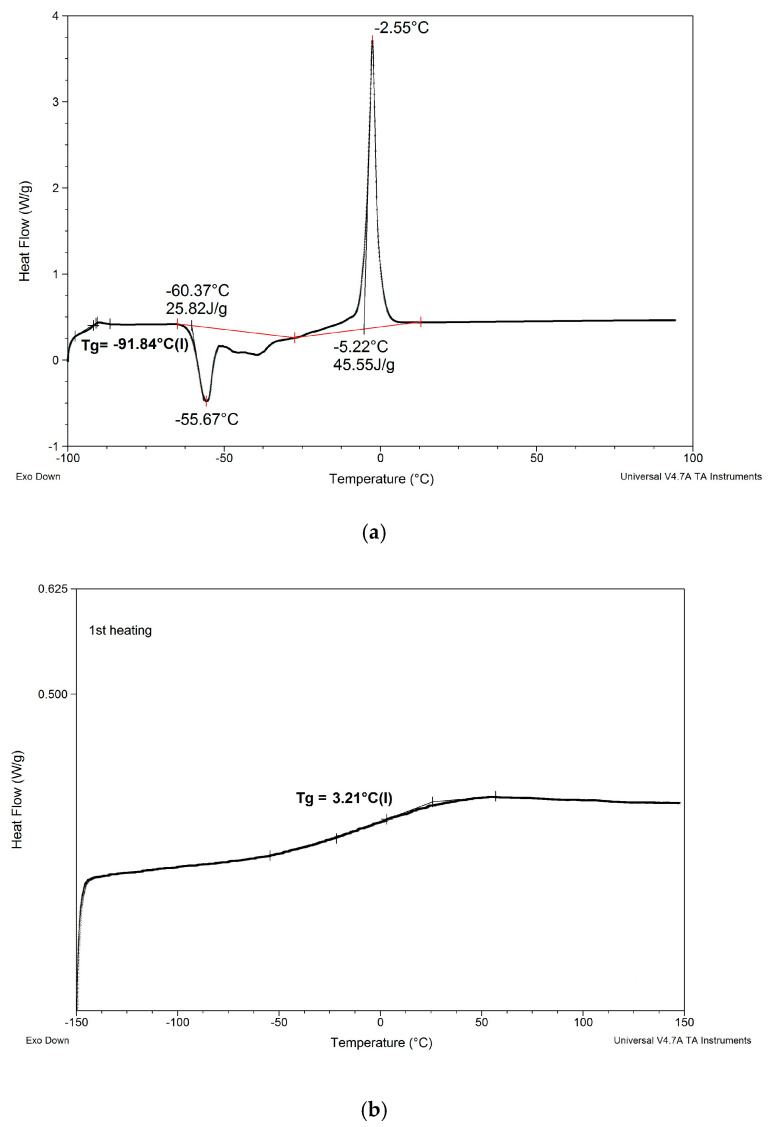
Comparison of DSC patterns of reactive IL 3 (**a**) and UV-cured reactive IL 3 (**b**).

Based on the results shown in Table 6 and Figure 7 and Figure 8, the following can be concluded:

(1) As in the case of modification with non-reactive ILs, replacing EC/DMC solvent with IL did not affect Tg of the silicone part (TgI) of the PSUR polymer constituting the body of the composite film. All composites except for K where 80% of PSUR prepolymer was replaced with reactive IL showed TgI values in the narrow range −125 to −129 °C. The increase in TgI in the case of composite K (see Figure 8) can be explained by the limited freedom of poly(dimethylsiloxane) chains in partly crosslinked PSUR polymer.

(2) As in the case of non-reactive ILs, none of the composites showed melting points of EC or ILs, which would remain in the composite film after the curing process.

(3) Replacement of 20% of the prepolymer with reactive IL resulted in a distinct increase in Tg of the PEO part of the PSUR polymer (TgII), which can be explained by the formation of a crosslinked structure that reduced the mobility of the PEO segments. However, this adverse effect totally disappeared when EC/DMC was replaced with non-reactive IL in composite M, and TgII of that composite was distinctly lower than TgII of composite G—see Figure 7.

(4) The type of anion in reactive IL seemed to influence somehow the TgII of the composite—the composite prepared with IL holding TFSI anion (IL 3) showed lower TgII than the composite holding TFI anion (IL 4).

(5) When the degree of crosslinking of the PSUR polymer was increased by increasing the reactive IL/prepolymer ratio, TgI increased (see item 1 above for explanation), but TgII did not change. However, and additional Tg (TgIII) appeared in the range +40 to +50 °C, which most probably corresponded to Tg of new polymer chains formed by polymerization of cations of reactive IL 3—see Figure 9, where DSC patterns of the reactive IL 3 before and after UV curing are compared. Nevertheless, it should be noted that Tg measured for the polymer produced from the same reactive IL was much lower (ca. +3 °C). This phenomenon can be explained by the formation of a kind of semi-IPN structure, where chains of polymerized reactive IL cations were embedded in the body of crosslinked PSUR, although some grafting of these chains on PSUR polymer could not be excluded.

### 3.5. Effect of Modification with ILs on Morphology of the PSUR-Based UV-Cured Composites

**Figure 10:**
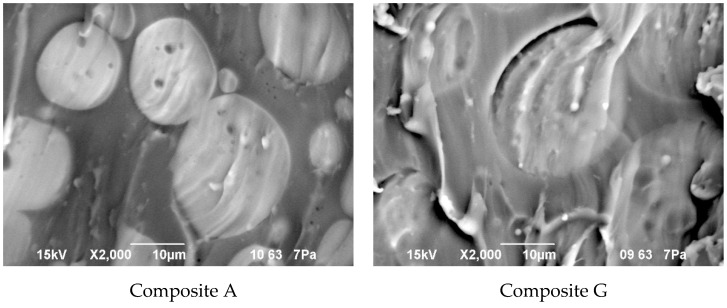
Morphology of the composite film prepared using EC/DMC as solvent (**Composite A**), and the composite film where that solvent was replaced with reactive IL 3 (**Composite G**).

**Figure 11:**
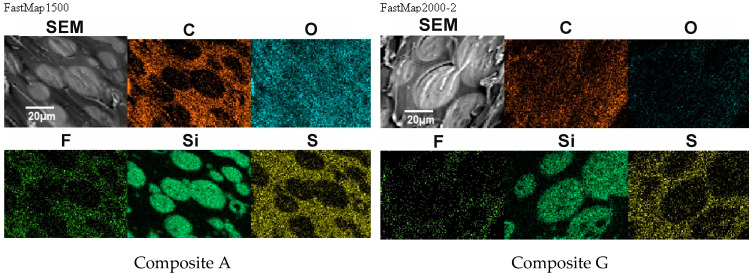
Distribution of elements in the composite film prepared using EC/DMC as solvent (**Composite A**) and the composite film where that solvent was replaced with reactive IL 3 (**Composite G**).

Morphology of the composite film prepared using EC/DMC as solvent for both prepolymer and lithium salt (A) and the composite film, where only solvent for prepolymer was replaced with reactive IL 3 (G), is presented in Figure 10, while the EDS images showing the distribution of specific elements (in particular Si originating from silicone part of the composite and F or S originating from lithium salt anion) are displayed in Figure 11.

It seems clear from Figure 10 that the morphology of the composite films prepared using EC/DMC solvent (A) and using that solvent and IL (G) did not differ much. In both cases it was a domain structure—silicone-rich domains of a few microns in size were embedded in the continuous phase. The only difference was in the phase separation level, which was slightly greater in the case of the composite film prepared using EC/DMC solvent only.

**Figure 12:**
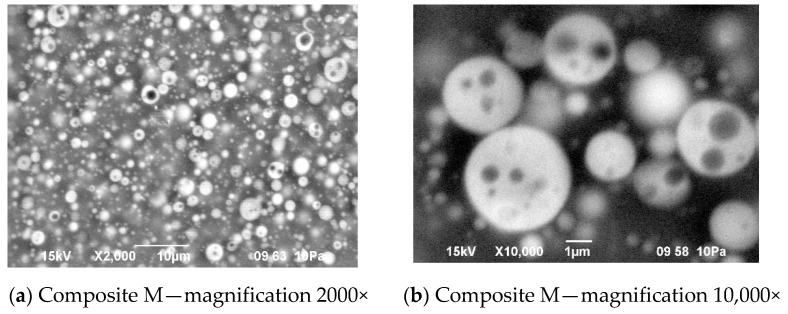
Morphology of the composite film M prepared using reactive IL (IL 3) to dissolve prepolymer (**a**) and non-reactive IL (IL 5) to dissolve lithium salt (**b**).

There was also no difference in distribution of elements in both composite films—see Figure 11. In both cases, the silicone part of the polymer formed the domains, while the lithium salt was placed only in the PEO-based phase. However, a different morphology was found for the composite film prepared in the same way as G, but with IL used not only for dissolving the prepolymer, but also for dissolving lithium salt (M). It means that the composite M was obtained without any other solvents except ILs—see Figure 12.

**Figure 13:**
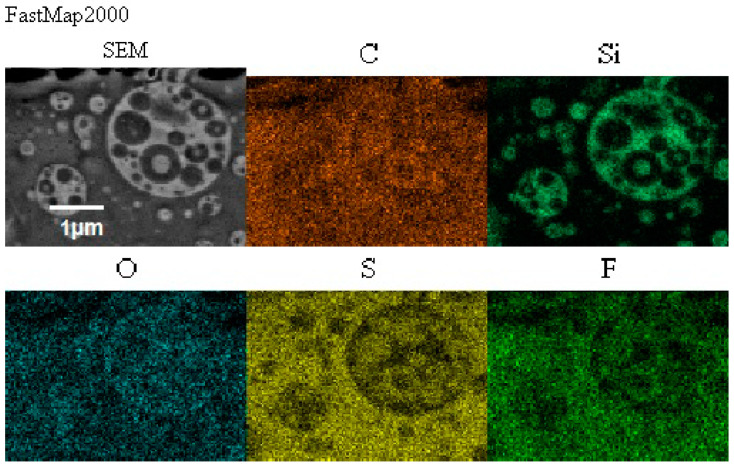
Distribution of elements in the composite film M prepared using reactive IL (IL 3) to dissolve prepolymer and non-reactive IL (IL 5) to dissolve lithium salt.

In this structure that was similar to the structure observed for composite films obtained using EC/DMC as solvent for both prepolymer and lithium salt [2] the silicone domains were much smaller (0.1–3 μ), and some of them contained also tiny domains of the PEO-based phase. It is worth noting that this composite showed reasonably good specific conductivity—in the range of 2 × 10^−3^ Scm^−1^ when DMC was used for wetting the film and surprisingly good specific conductivity—in the range of 1 × 10^−4^ Scm^−1^ when IL was used for that purpose. The reason for good specific conductivity for this composite film could be that the IL acted as compatibilizer for silicone and PEO phases—perhaps a kind of complex of IL cation with PEO could be formed. Such interaction of IL cations with oxygen atoms of PEO in gel polymer electrolyte of much simpler structure (high MW PEO + Li salt + IL) also designed for lithium ion batteries was proved earlier by the other authors who analyzed its FTIR spectrum [41]. Distribution of elements in our selected composite film presented in Figure 13 seems to confirm that suggestion, since it is clear that IL was concentrated in the PEO phase and the silicone phase formed large separate domains. Nevertheless, these results anticipated that the morphology of the PSUR-based UV-cured composites might have some effect on their specific conductivity, while in general the ionic conduction behavior was primarily governed by the diffusion of ions within the solvent. Obviously, the main effect of IL was to increase the amorphous phase content, leading to considerable increase in PEO chain flexibility [41,79,80]. In the PSUR-based UV-cured composites described in our paper, this effect was even enhanced by the presence of a silicone phase that also decreased significantly the crystallinity level of PEO, as confirmed by us earlier [1], so the PEO melting point was actually absent in DSC patterns—see Figure 5, Figure 7, and Figure 8.

### 3.6. Preliminary Testing of the Selected PSUR-Based UV-Cured Composite Film as Separator for Lithium Ion Batteries

**Figure 14:**
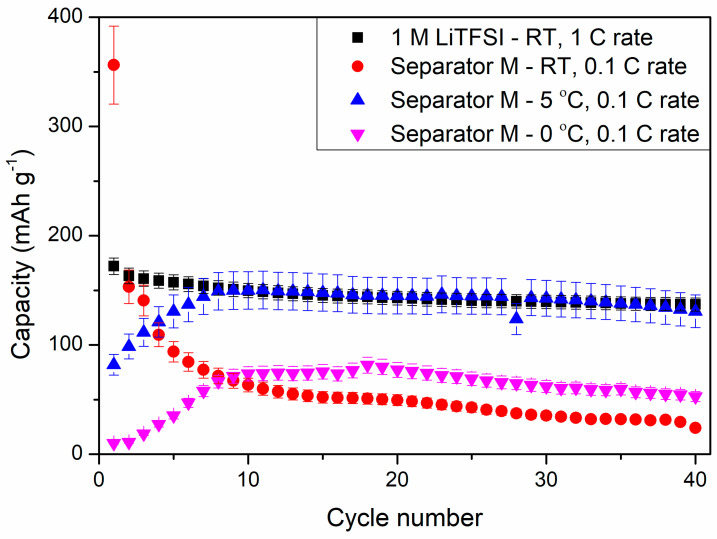
Cyclability tests of PSUR-based UV-cured composite film M applied as a conductive separator and (for comparison) 1 M LiTFSI liquid electrolyte in lithium-ion cells at different temperatures.

Figure 14 shows the results of cyclability tests of the selected composite (M) obtained in our study at various temperatures and a reference sample at 1 C rate at room temperature (RT). The cell with LiTFSI as liquid electrolyte showed discharge capacity of 172 ± 8 mAh g^−1^ at the first cycle, which slowly decreased to 137 ± 6 mAh g^−1^ at the 40th discharge process. The replacement of liquid electrolyte with composite film M resulted in a large specific capacity at the first discharge process, reaching 356 ± 36 mAh g^−1^, which decreased to 153 ± 15 mAh g^−1^ in the next cycle. Such a large irreversible capacity could result from composite M side reactions with metallic lithium. Moreover, the specific capacity of the cell was continuously decreasing to 24 ± 2 mAh g^−1^ in the 40th cycle at RT. This phenomenon might suggest a continuous degradation of the composite material during cycling at RT.

However, after lowering the ambient temperature, the electrochemical performance of the cells changed drastically. At both 5 °C and 0 °C, the capacity of the cells was increasing for the first 10 cycles, reaching 150 ± 17 and 74 ± 7 mAh g^−1^ at 5 °C and 0 °C, respectively. For comparison, the reference sample showed a specific capacity of 150 ± 7 mAh g^−1^ at RT and 1 C current rate. Lowering the ambient temperature could hinder the side reactions of composite M and increase its stability in the Li-ion cell. During the first three cycles, the cells were operating as one could expect, with the performances following the trend of RT > 5 °C > 0 °C. However, at RT, the composite suffered from side reactions, which could be responsible for gel electrolyte degradation and deterioration of cell electrochemical properties. The cell performances at 5 and 0 °C showed increased stability, which could be related to better chemical and mechanical durability of the separator throughout the experiment and hindered degradation of the composite. However, this phenomenon needs further investigation.

**Figure 15:**
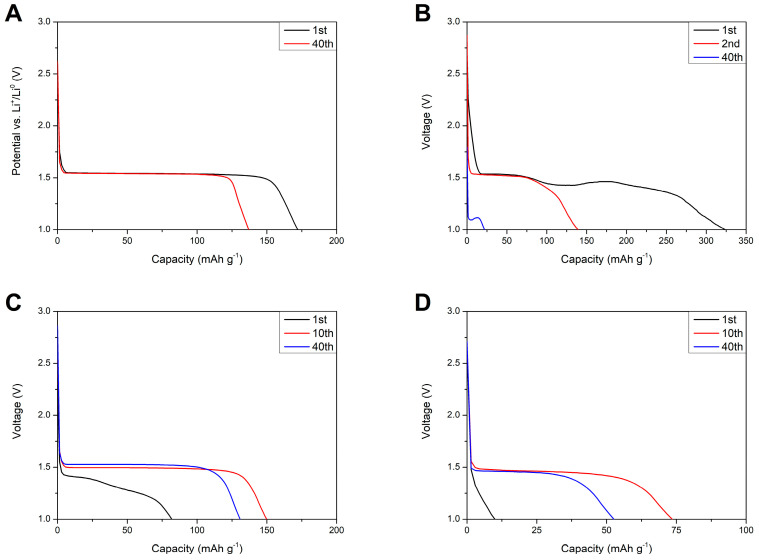
Discharge profiles of Li_4_Ti_5_O_12_ electrodes in different environments: 1 M LiTFSI at 1 C rate and RT (**A**) and composite M separator at 0.1 C rate and at different temperatures: RT (**B**), 5 °C (**C**), and 0 °C (**D**).

Figure 15 shows discharge profiles of lithium–titanium oxide electrodes in different environments. For the reference sample (Figure 15A) one can see a stable potential plateau at 1.55 V (vs. Li^+^/Li^0^) originating from the two-phase reaction—Equation (3).

Li_4_Ti_5_O_12_ + 3Li^+^ = Li_7_Ti_5_O_12_ (3)

Replacing the liquid electrolyte with gel polymer separator resulted in a change in the shape of voltage profiles. At RT, the discharge profile was extended past the regular 1.55 V plateau, which might be caused by side reactions of the polymer film with the lithium counter electrode and responsible for irregularities in the cell voltage. After 40 charge/discharge cycles, the voltage plateau was almost negligible and moved to lower voltages of ca. 1.1 V, suggesting increased polarization in the cells as a result of continuous polymer degradation. At 5 °C, the first discharge profile still consisted of some irregularities, which might have originated from side reactions of the separator and metallic lithium. After the 10th cycle, the shape of the curve changed to a regular two-phase reaction of Li_4_Ti_5_O_12_ with lithium ions and remained stable for the remaining charge/discharge cycles. At 0 °C, the first discharge profile showed no plateau and purely capacitive characteristics of the electrode. After the 10th cycle, the voltage profile transformed to a two-phase faradaic reaction and, like in the case of the cells examined at 5 °C, remained stable for the next charge/discharge cycles.

**Figure 16:**
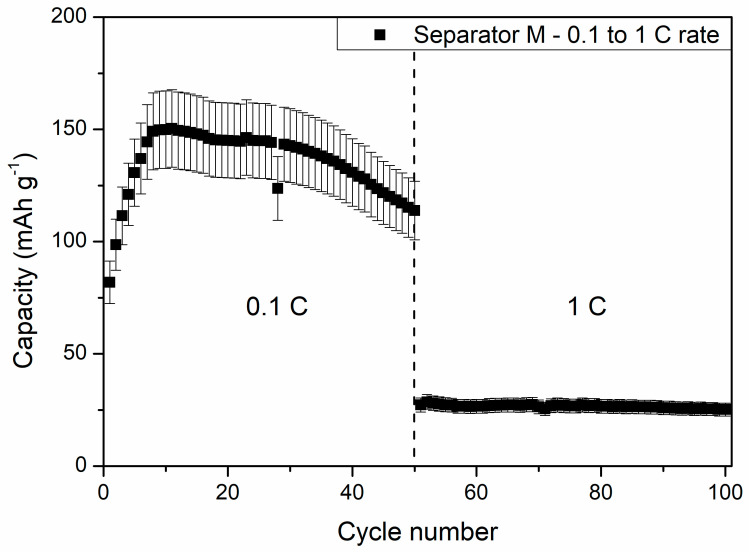
The effect of further cycling of the cell with composite film M applied as a conductive separator at 5 °C and 1 C current rate—exceptionally stable work of the battery can be noted.

The slow increase in the capacity of cells at 5 °C and 0 °C could be a result of wettability increase of the separator by DMC addition, which assisted in ion migration from/into electrodes and the separator in consecutive cycles. After 40 charge/discharge cycles, the cells delivered 131 ± 15 and 53 ± 5 mAh g^−1^ at 5 °C and 0 °C, respectively, showing increased stability while compared to being charged/discharged at RT. One can see that the specific capacities of the cells measured at 5 °C was similar to the reference liquid electrolyte cell measured at 1 C current rate at RT. Because of its good electrochemical performance, an additional 50 cycles at 1 C rate and 5 °C were performed for the cells with the PSUR separator, which are presented in Figure 16. As can be seen from Figure 16, the capacity was not satisfactory (only 25 mAh/g while with 0.1 C current it was 115 mAh/g), but the electrochemical performance of the battery was stable, unlike when the battery was tested at 25 °C.

From Figure 16, one can also see a drastic capacity drop after switching the discharge current rate from 0.1 C to 1 C. The specific capacity dropped from 114 ± 13 to 27 ± 3 mAh g^−1^ after increasing the current rate tenfold. This effect might be the result of insufficient ionic transport between the composite film M and Li_4_Ti_5_O_12_ material grains, causing large polarization of the electrodes, hindering full completion of the electrochemical reaction. Despite the large polarization effect, the stability of the cells remained high, and at the end of the measurement, the cell delivered a specific capacity of 25 ± 3 mAh g^−1^.

## 4. Conclusions

It appeared possible to obtain EC/DMC solvent-free composite films potentially suitable as separators for lithium ion batteries showing specific conductivity in the range of 10^−3^–10^−4^ Scm^−1^ by in-situ modification of UV-cured PSUR containing lithium salts with ILs. Like the UV-cured PSUR composites containing EC/DMC solvent described in our earlier paper [2], the IL-modified composites investigated in the current study also showed two-phase morphology, where the silicone part of the composite formed well-defined domains embedded in the continuous PEO-based phase. The size of the silicone domains was much bigger (ca. 10 μ) when non-reactive ILs were applied and much smaller (0.1–3 μ) when reactive UV-curable ILs were used, and some of these smaller domains contained tiny drops of PEO-based phase. This phenomenon resulted in better conductivity of the composite film and could be explained by the IL acting as compatibilizer for the silicone phase and PEO-based phase. Such suggestion was supported with DSC investigations, which revealed that in these composites, the Tg of the PEO-based phase was much lower (<−40 °C) than the Tg of the composites modified with the IL/EC/DMC mixture (ca. +3 °C). It is interesting that in the same time, Tg of the silicone phase did not change much and remained at the level of −125 °C. The results of preliminary tests of applying the UV-cured PSUR composite film modified with a mixture of reactive IL, i.e., diallyldimethylammonium bis(trifluoromethylsunfonyl)imide and non-reactive IL, i.e., 1-butyl-1-methylpyrrolidinium bis(trifluoromethylsulfonyl)imide as separator for experimental lithium ion battery, showed that this approach is quite promising. The PSUR-based UV-cured composite film that could play the role of gel polymer electrolyte and separator in Li-ion cells showed good electrochemical stability at low temperatures. The new innovative lithium ion battery developed based on technology described in our paper was patented [81], though it is clear that further studies are needed until this development may lead to a new class of lithium ion batteries produced without any flammable solvent.

## Figures and Tables

**Table 1:** Names, substantial properties, and structures of ionic liquids (ILs) used in the study.

	Name and Properties	Structure
1.	Didecyldimethylammonium bis(trifluoromethylsulfonyl)imide (H_2_O content = 0.05%; Cl content < 0.01%)	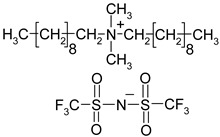
2.	Benzalkonium bis(trifluoromethylsulfonyl)imide (H_2_O content = 0.05%; Cl content < 0.01%)	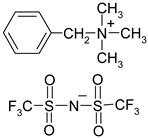
3.	Diallyldimethylammonium bis(trifluoromethylsulfonyl)imide (H_2_O content = 0.02%; Cl content < 0.01%)	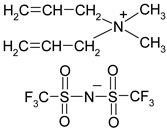
4.	Diallyldimethylammonium trifluoromethanesulfonate (H_2_O content = 0.07%; Cl content < 0.12%)	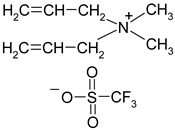
5.	1-butyl-1-methylpyrrolidinium bis(trifluoromethylsulfonyl)imide (H_2_O content ≤ 0.04%; Cl content ≤ 0.005%)	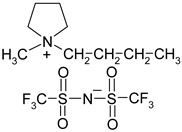
6.	1-ethyl-3-methylimidazolium trifluoromethanesulfonate (H_2_O content ≤ 0.02%)	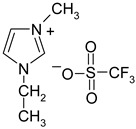

**Table 2:** Melting points, cold crystallization temperatures, and glass transition temperatures (Tgs) of ILs applied in the study. Names of ILs correspond to numbers in Table 1.

Name of IL	Melting Point °C	Cold Crystallization Temperature °C	Tg °C	Comments
IL 1	ca. −65	None	−79.96	Diffused melting point
IL 2	−53.16	None	−55.85	-
IL 3	−2.55	−55.67	−91.84	Very sharp melting point
IL 3 (after UV curing)	None	None	+3.21	-
IL 4	−5.27	−51.64	−92.27	Sharp melting point
IL 5	−13.79	−53.37	−90.44	Sharp melting point
IL 6	−18.87 and −7.35	−44.08	−85.10	Sharp melting and freezing points

**Table 3:** Compositions of the PSUR-based UV-cured composites containing non-reactive ILs obtained in the study and the results of specific conductivity determinations. Composite prepared without IL (A) was included for comparison. Composite N without any solvent and lithium salt was also prepared for Tg determinations only.

Name of the Composite	Lithium Salt Anion	Solvent for Lithium Salt	Mass Proportion		Compound for Wetting the Film	Specific Conductivity Scm^−1^ × 10^−4^
			Li Salt to Prepolymer	Solvent or/and IL to Prepolymer		
A	TFSI	EC/DMC = 1/2	1/5	4/5	EC/DMC = 1/2	7.47
B	TFSI	IL 5	1/5	4/5	IL 5	0.63
D	TFSI	IL 1	1/5	4/5	IL 1	0.02
D-D	TFSI	IL 1	1/5	4/5	DMC	1.47
E	TFSI	IL 2/DMC =1/2	1/5	4/5	DMC	3.11
E-C	TFSI	IL 2/DMC = 1/2	1/5	4/5	IL 2	0.01
F	TFSI	IL 5/DMC =1/2	1/5	4/5	DMC	3.92
F-C	TFSI	IL 5/DMC = 1/2	1/5	4/5	IL 5	0.14
I	TFI	IL 6/DMC = 1/2	1/5	4/5	DMC	4.26
I-C	TFI	IL 6 DMC = 1/2	1/5	4/5	IL 6	0.66
N	-	-	-	-	-	-

**Table 4:** Results of Tg determinations obtained for PSUR-based UV-cured composites containing non-reactive ILs. Composites prepared without IL (A) and without any solvent and lithium salt (N) were included for comparison.

Name of the Composite	TgI °C	TgII °C	TgIII °C	Comments
A	−125.25	−11.32	None	-
B	−124.29	−46.28	+65.11	TgIII—very weak signal
E	−123.43	−23.47	None	-
F	−125.96	−15.02	+49.56 to 82.56	TgIII—very weak signal
I	−129.41	−26.0	+58.49 to 85.69	TgIII—very weak signal
N	−126.93	−27.47	None	-

**Table 5:** Compositions of the PSUR-based UV-cured composites containing reactive ILs obtained in the study and the results of specific conductivity determinations. Composite prepared without IL (A) was included for comparison. Composite N without any solvent and lithium salt was also prepared for Tg determinations only.

Name of the Composite	Lithium Salt Anion	Solvent for Lithium Salt	Mass Proportion		Compound for Wetting the Film	Specific Conductivity Scm^−1^ × 10^−4^
			Li Salt to Prepolymer	Solvent or/and IL to Prepolymer		
A	TFSI	EC/DMC = 1/2	1/5	4/5	EC/DMC = 1/2	7.47
G	TFSI	IL 3/(EC/DMC = 1/2) = 1/3.5	1.5/4	4.5/4	EC/DMC = 1/2	10.8
G-C	TFSI	IL 3/(EC/DMC = 1/2) = 1/3.5	1.5/4	4.5/4	IL 3	0.16
G-D	TFSI	IL 3/(EC/DMC = 1/2) = 1/3.5	1.5/4	4.5/4	DMC	2.03
H	TFI	IL 4/(EC/DMC = 1/2) = 1/4.2	0.8/4	5.2/4	EC/DMC = 1/2	3.68
K	TFSI	IL 3/(EC/DMC = 1/2) = 4/3.5	6/4	30/4	IL 3	0.14
K-D	TFSI	IL 3/(EC/DMC = 1/2) = 4/3.5	6/4	30/4	DMC	3.23
M	TFSI	IL 3/IL 5 = 1/4	1/4	5/4	IL 5	1.02
M-D	TFSI	IL 3/IL 5 = 1/4	1/4	5/4	DMC	18.7
M-CS	TFSI	IL 3/IL 5 = 1/4	1/4	5/4	1M IL 5 in 1M Li TFSI	0.60
N	-	-	-	-	-	-

**Table 6:** Results of Tg determinations obtained for PSUR-based UV-cured composites containing reactive ILs. Composites prepared without IL (A) and without any solvent and lithium salt (N) were included for comparison.

Name of the Composite	TgI °C	TgII °C	TgIII °C	Comments
G	−125.48	+3.09	None	-
H	−129.00	+10.93	+32.87–56.06	TgIII—very weak signal
I	−129.41	−26.20	+58.49–85.69	TgIII—very weak signal
K	−119.35	+11.04	None	-
M	−125.53	−46.52	+124.13	TgIII—very weak signal
N	−126.93	−27.47	None	-

## References

[1] Kozakiewicz J., Przybylski J., Sylwestrzak K. (2016). Silicone-urethane membranes for lithium batteries. Part 1. Moisture-cured poly(siloxaneurethane-urea) elastomers containing polyethylene oxide (PEO) segments—Synthesis and characterization as potential membrane materials. Polym. Adv. Technol..

[2] Kozakiewicz J., Przybylski J., Sylwestrzak K. (2019). Synthesis and characterization of UV-cured poly(siloxane-urethane) elastomers containing polyethylene oxide segments—As potential membrane materials. Polimery.

[3] Song M., Cho J., Cho B.W., Rhee H. (2002). Characterization of UV-cured gel polymer electrolytes for rechargeable lithium batteries. J. Power Sources.

[4] Song M., Kim Y., Kim Y.T., Cho B., Popov N., Rhee H. (2003). Thermally stable gel polymer electrolytes. J. Electrochem. Soc..

[5] Gerbaldi C. (2010). All-solid-state lithium-based polymer cells for high-temperature applications. Ionics.

[6] Nair J.R., Gerbaldi C., Meligrana G., Bongiovanni R., Bodoardo S., Penazzi N., Reale P., Gentili V. (2008). UV-cured methacrylic membranes as novel gel–polymer electrolyte for Li-ion batteries. J. Power Sources.

[7] Gerbaldi C., Nair J.R., Meligrana G., Bongiovanni R., Bodoardo S., Penazzi N. (2009). Highly ionic conducting methacrylic-based gel polymer electrolytes by UV-curing technique. J. Appl. Electrochem..

[8] Porcarelli L., Gerbaldi C., Bella F., Nair J.R. (2016). Super soft all-ethylene oxide polymer electrolyte for safe all-solid lithium batteries. Sci. Rep..

[9] Kil E.H., Choi K.H., Ha H.J., Xu S., Rogers J.H., Kim M.R., Lee Y.G., Kim K.M., Cho K.Y., Lee S.Y. (2013). Imprintable, bendable and shape-conformable polymer electrolytes for versatile-shaped lithium-ion batteries. Adv. Mater..

[10] Imperiyka M., Ahmad A., Hanifah S.A., Rahman M.Y.A. (2013). Potential UV-curable poly(glycidyl methacrylate-co-ethyl methacrylate)- based solid polymer electrolyte for lithium ion battery application. Int. J. Electrochem. Sci..

[11] Oh B., Jung W.I., Kim D.W., Rhee H.W. (2002). Preparation of UV-curable gel polymer electrolytes and their electrochemical properties. Bull. Korean Chem. Soc..

[12] Gerbaldi C., Nair J.R., Meligrana G., Bongiovanni R., Bodoardo S., Penazzi N. (2010). UV-curable siloxane-acrylate gel copolymer electrolytes for lithium-based battery applications. Electrochim. Acta.

[13] Destro M., Gerbaldi C., Bella F., Nair J.R. (2015). Siloxane diacrylate-based all solid polymer electrolytes for lithium batteries. Int. J. Membr. Sci. Technol..

[14] Kim C.S., Kim B.H., Kim K. (1999). Synthesis and characterization of polyether urethane acrylate-LiCF_3_SO_3_-based polymer electrolytes by UV-curing in lithium batteries. J. Power Sources.

[15] Ugur M.H., Kihc H., Berkem M.L., Gungor A. (2014). Synthesis by UV-curing and characterization of polyurethane acrylate-lithium salts-based polymer electrolytes in lithium batteries. Chem. Pap..

[16] Susan M.A.B.H., Kaneko T., Noda A., Watanabe M. (2005). Ion gels prepared by in situ radical polymerization of vinyl monomers in an ionic liquid and their characterization as polymer electrolytes. J. Am. Chem. Soc..

[17] Nakagawa H., Izuchi S., Kuwana K., Nukuda T., Aichara Y. (2003). Liquid and polymer gel electrolytes for lithium batteries composed of room-temperature molten salt doped by lithium salt. J. Electrochem. Soc..

[18] Ohno H. (2005). Electrochemical Aspects of Ionic Liquids.

[19] Ye Y.S., Rick J., Hwang B.J. (2013). Ionic liquid polymer electrolytes. J. Mater. Chem. A.

[20] Karuppassamy K., Theerthagiri J., Vikraman G., Yim C.J., Hussain S., Sharma R., Maiyalagan T., Qin J., Kim H.S. (2020). Ionic liquid-based electrolytes for energy storage devices; A bief review of their limits and applications. Polymers.

[21] Gerbaldi C., Nair J.R., Ahmad S., Meligrana G., Bongiovanni R., Bodoardo S., Penazzi N. (2010). UV-cured polymer electrolytes encompassing hydrophobic room temperature ionic liquid for lithium batteries. J. Power Sources.

[22] Nair J.R., Porcarelli R., Bella F., Gerbaldi C. (2015). Newly elaborated multipurpose polymer electrolyte encompassing RTILs for smart energy-efficient devices. ACS Appl. Mater. Interfaces.

[23] Woo H.S., Son H., Min J.Y., Rhee J., Lee H.T., Kim D.W. (2020). Ionic liquid-based gel polymer electrolyte containing zwitterion for lithium oxygen batteries. Electrochim. Acta.

[24] Porcarelli K., Manojkumar K., Sardon H., Llorente O., Shaplov A.S., Vijayakrishna K., Gerbaldi C., Mecerreyes D. (2017). Single ion conducting polyemnr electrolytes based on versatile polyurethanes. Electrochim. Acta.

[25] Chaudoy V., Tran Van F., Deschamps M., Ghamouss F. (2017). Ionic liquids in a polyethylene oxide cross-linked gel polymer as an electrolyte for electrical double layer capacitor. J. Power Sources.

[26] Rupp B., Schmuck M., Balducci A., Winter M., Kern W. (2008). Polymer electrolyte for lithium batteries based on photochemically crosslinked poly(ethylene oxide) and ionic liquid. Euro. Polym. J..

[27] Stępniak I., Andrzejewska E., Debna A., Galiński M. (2014). Characterization and application of N-methyl-N-propylpiperidinium bis (trifluormethhanesulfonyl)imide ionic liquid-based gel polymer electrolyte prepared in situ by photopolymerization method in lithium batteries. Electrochim. Acta.

[28] Sigma Aldrich and Fluka Leaflet: “Enabling Technologies—Ionic Liquids”, Chem. Files vol. 5 No. 6. https://www.yumpu.com/en/document/read/6902632/vol-5-no-6-on-ionic-liquids-sigma-aldrich.

[29] Welton T. (1999). Room temperature ionic liquids. Solvents for synthesis and catalysis. Chem. Rev..

[30] Lu J., Yan F., Texter J. (2009). Advanced applications of ionic liquids in *polymer science*. Progr. Polym. Sci..

[31] Keskin S., Kayrak-Talay D., Akman U., Hortacsu O. (2007). A review of ionic liquids towards supercritical fluid applications. Supercrit. Fluids.

[32] Pernak J., Walkiewicz F., Maciejewska M., Zaborski M. (2010). Ionic liquids as vulcanization accelerators. Ind. Eng. Chem. Res..

[33] Tsuda T., Hussey C.L. (2007). Electrochemical applications of room-temperature ionic liquids. Electrochem. Soc. Interface.

[34] Silvester D.S., Compton G. (2006). Electrochemistry in room temperature ionic liquids; A review and some possible applications. Z. Phys. Chem..

[35] Hasanzadeh M., Shadjou N., Eskandani M., de la Guardia M. (2012). Room-temperature ionic liquid-based electrochemical nanobiosensors. TrAC Trends Anal. Chem..

[36] Paul A., Muthukumar S., Prasad S. (2020). Review—Room temperature ionic liquids for electrochemical applications with special focus on gas sensors. J. Electrochem. Soc..

[37] Kim G.T., Appetecchi G.B., Carewska M., Joost M., Balducci A., Winter M., Passerini S. (2010). UV-crosslinked, lithium conducting ternary polymer electrolytes containing ionic liquids. J. Power Sources.

[38] Shaplov A.S., Marcilla R., Mecerreyes D. (2015). Recent advances in innovative polymer electrolytes based on poly(ionic liquid)s. Electrochim. Acta.

[39] Lewandowski A., Świderska-Mocek A. (2009). Ionic liquids as electrolytes for Li-ion batteries—An overview of electrochemical studies. J. Power Sources.

[40] Kim G.T., Passerini S., Carewska M., Appetecchi G.B. (2018). Ionic liquid-based electrolyte membranes for medium-high temperature lithium polymer batteries. Membranes.

[41] Polu A.R., Rhee H.W. (2017). Ionic liquid doped PEO-based solid polimer electrolytes for lithium ion polymer batteries. Int. J. Hydrog. Energy.

[42] Diaw M., Chagnes A., Carre B., Willmann P., Lemordant D. (2005). Mixed ionic liquid as electrolyte fpr lithium batteries. J. Power Sources.

[43] Gao X., Wu F., Mariani A., Passerini S. (2019). Concentrated ionic-liqid-based electrolytes for high voltage lithium batteries with improved performance at room temperature. ChemSusChem.

[44] Elia G.A., Ulissi U., Jeong S., Passerini S., Hassoun J. (2017). Exceptional long-life performance of lithium-ion batteries using ionic liquid-based electrolytes. Energy Environ. Sci..

[45] Francis C.F.J., Kyratzis Y.R., Best A.S. (2020). Lithium-ion battery separators for ionic-liquid electrolytes: A Review. Adv. Mater..

[46] Kirchhofer M., von Zamory J., Paillard E., Passerini S. (2014). Separators for Li-ion and Li-metal battery including ionic liquid based electrolytes based on the TFSI- and FSI- anions. Int. J. Mol. Sci..

[47] Xiang Y., Li J., Lei J., Liu D., Xie Z., Qu D., Li K., Deng T., Tang H. (2016). Advanced separators for lithium-ion and lithium-sulfur batteries: A review of recent progress. ChemSusChem.

[48] Ueki T., Watanabe M. (2008). Macromolecules in ionic liquids; progress, challenges and opportunities. Macromolecules.

[49] Eftekhari A., Chen P. (2016). Different roles of ionic liquids in lithium batteries. J. Power Sources.

[50] Kumar Y., Hashmi S.A., Pandey G.P. (2011). Lithium ion transport and ion–polymer interaction in PEO based polymer electrolyte plasticized with ionic liquid. Solid State Ion..

[51] Park M., Zhang X., Chung M., Less G.B., Sastry A.M. (2010). A review of conduction phenomena in Li-ion batteries. J. Power Sources.

[52] Oltean G., Plylahan L., Ihrfors C., Wei W., Xu C., Edstroem K., Nyholm L., Johansson P., Gustaffson T. (2018). Towards Li-ion batteries operating at 80 °C: Ionic liquid versus conventional liquid electrolytes. Batteries.

[53] Marsh K.N., Boxall J.A., Lichtenthaler R. (2004). Room temperature ionic liquids and their mixtures—A review. Fluid Phase Equilibria.

[54] Patel R., Seo J.A., Koh J.H., Kim J.H., Kang Y.S. (2011). Dye-sensitized solar cells employing amphiphilic poly(ethylene glycol) electrolytes. J. Photochem. Photobiol. A Chem..

[55] Kim G.T., Appetecchi G.B., Alessandrini F., Passerini S. (2007). Solvent-free, PYR1ATFSI ionic liquid-based ternary polymer electrolyte systems. J. Power Sources.

[56] Abitelli E., Ferrari S., Quartarone E., Mustarelli P., Magistris A., Fagnoni M., Albini A., Gerbaldi C. (2010). Polyethylene oxide electrolyte membranes with pyrrolidinium-based ionic liquids. Electrochim. Acta.

[57] Shin J.H., Henderson W.A., Passerini S. (2005). PEO-Based Polymer Electrolytes with Ionic Liquids and Their Use in Lithium Metal-Polymer Electrolyte Batteries. J. Electrochem. Soc..

[58] Cheng H., Zhu C., Huang B., Lu M., Yong Y. (2007). Synthesis and electrochemical characterization of PEO-based polymer electrolytes with room temperature ionic liquids. Electrochim. Acta.

[59] Singh P.K., Kim K.W., Rhee H.W. (2009). Development and characterization of ionic liquid doped solid polymer electrolyte membranes for better efficiency. Synth. Metals.

[60] Singh P.K., Kim K.W., Rhee H.W. (2008). Electrical, optical and photoelectrochemical studies on a solid PEO-polymer electrolyte doped with low viscosity ionic liquid. Electrochem. Commun..

[61] Dobbelin M., Azcune I., Bedu M., Ruiz de Luzuriaga A., Genua A., Jovanovski V., Cabanero G., Odriozola I. (2012). Synthesis of pyrrolidinium-based poly(ionic liquid) electrolytes with poly(ethylene glycol) side chains. Chem. Mater..

[62] Zygadło-Monikowska E., Florjańczyk Z., Służewska K., Ostrowska J., Langwald N., Tomaszewska A. (2010). Lithium conducting ionic liquids based on lithium borate salts. J. Power Sources.

[63] Stępniak I., Andrzejewska E. (2009). Highly conductive ionic liquid based ternary polymer electrolytes obtained by in situ photopolymerization. Electrochim. Acta.

[64] Matsumoto K., Talukdar B., Endo T. (2011). Methacrylate-based ionic liquid: Radical polymerization/copolymerization with methyl methacrylate and evaluation of molecular weight of the obtained homopolymers. Polym. Bull..

[65] Eshetu G.G., Mecerreyes D., Forsyth M., Zhang H., Armand M. (2019). Polymeric ionic liquids for lithium-based rechargeable batteries. Mol. Syst. Des. Eng..

[66] Yang G., Song Y., Wang Q., Zhang L., Deng L. (2020). Review of ionic liquids-containing polymer/inorganic hybrid electrolytes for lithium batteries. Mater. Des..

[67] Braga M.H., Grundish N.S., Murchison A.J., Goodenough J.B. (2017). Alternative strategy for a safe rechargeable battery. Energy Environ. Sci..

[68] Zhou D., Liu R., Zhang J., Qi X., He Y.-B., Li B., Yang Q.-H., Hu Y.-S., Kang F. (2017). In situ synthesis of hierarchical poly(ionic liquid)-based solid electrolytes for high-safety lithium-ion and sodium-ion batteries. Nano Energy.

[69] Ma F., Zhang Z., Yan W., Ma X., Sun D., Jin Y., Chen X., He K. (2019). Solid polymer electrolyte based on polymerized ionic liquid for high performance all-solid-state lithium-ion batteries. ACS Sustain. Chem. Eng..

[70] Sato T., Marukane S., Narutomi T., Akao T. (2007). High rate performance of a lithium polymer battery using a novel ionic liquid polymer composite. J. Power Sources.

[71] Nykaza J.R., Savage A.M., Pan Q., Wang S., Beyer F.L., Tang M.H., Li C.Y., Elabd Y.A. (2016). Polymerized ionic liquid diblock copolymer as solid-state electrolyte and separator in lithium-ion battery. Polymer.

[72] D’Angelo A.J., Panzer M.J. (2018). Decoupling the ionic conductivity and elastic modulus of gel electrolytes: Fully zwitterionic copolymer scaffolds in lithium salt/ionic liquid solutions. Adv. Energy Mater..

[73] Kozakiewicz J., Przybylski J., Sylwestrzak K. (2015). A Method of Making an Electroconductive Composite.

[74] Kozakiewicz J., Cybulski J., Przybylski J., Sylwestrzak K., Wisniewska A. (2015). A Method of Making an Electroconductive Composite.

[75] Katritzky A.R., Lomaka A., Petrukhin R., Jain R., Karelson M., Visser A.E., Rogers R.D. (2002). Room temperature ionic liquids and their mixtures—A review. J. Comp. Info. Comp. Sci..

[76] Liu L., Zheng Z., Gu C., Wang X. (2010). The poly(urethane-ionic liquid)/multiwalled carbon nanotubes composites. Compos. Sci. Technol..

[77] Appetecchi G.B., Kim G.T., Montanino M., Alessandrini F., Passerini S. (2011). Room temperature lithium polymer batteries based on ionic liquids. J. Power Sources.

[78] Gray F.M. (1991). Solid Polymer Electrolytes: Fundamentals and Technological Applications.

[79] Kharel A., Lodge T.P. (2019). Effect of ionic liquid components on coil dimensions of PEO. Macromolecules.

[80] Kharel A., Lodge T.P. (2017). Coil dimensions of poly(ethylene oxide) in an ionic liquid by small-angle neutron scattering. Macromolecules.

[81] Hamankiewicz B., Czerwiński A., Krajewski M., Michalska M., Lipińska L., Kozakiewicz J., Przybylski J., Sylwestrzak K., Sarna W. (2017). Lithium-Ion Cell.

